# Health-Related Quality of Life in Radiologically Isolated Syndrome Resembles Relapsing–Remitting Multiple Sclerosis

**DOI:** 10.3390/jcm15062184

**Published:** 2026-03-13

**Authors:** Julián Benito-León, María Díez-Cirarda, Mariano Ruiz-Ortiz, Yolanda Aladro, Constanza Cuevas, Ángela Domingo-Santos, Victoria Galán Sánchez-Seco, Andrés Labiano-Fontcuberta, Ana Gómez-López, Paula Salgado-Cámara, Lucienne Costa-Frossard, Enric Monreal, Susana Sainz de la Maza, Jorge Matías-Guiu, Paloma Montero-Escribano, María Luisa Martínez-Ginés, Lucía Ayuso-Peralta, Jordi A. Matías-Guiu

**Affiliations:** 1 Department of Neurology, 12 de Octubre University Hospital, 28041 Madrid, Spain; mariano.ruiz.ortiz@gmail.com (M.R.-O.); adomingosantos@hotmail.com (Á.D.-S.); vickyg_s@hotmail.com (V.G.S.-S.); gandhilabiano@hotmail.com (A.L.-F.); anagmlp@gmail.com (A.G.-L.); paula.salgado.camara@gmail.com (P.S.-C.); 2 Instituto de Investigación Sanitaria Hospital 12 de Octubre (imas12), 28041 Madrid, Spain; 3 Department of Medicine, Faculty of Medicine, Complutense University, 28040 Madrid, Spain; matiasguiu@gmail.com (J.M.-G.); jordimatiasguiu@hotmail.com (J.A.M.-G.); 4 Network Center for Biomedical Research in Neurodegenerative Diseases (CIBERNED), 28029 Madrid, Spain; 5 Department of Neurology, Hospital Universitario Clínico San Carlos, Health Research Institute “San Carlos” (IdISCC), 28040 Madrid, Spain; mdcirarda@salud.madrid.org (M.D.-C.); constanzaece@gmail.com (C.C.); pmontero84@gmail.com (P.M.-E.); 6 Department of Neurology, Getafe University Hospital, 28905 Getafe, Spain; yolanda.aladro@salud.madrid.org; 7 Faculty of Biomedical and Health Sciences, European University of Madrid, 28670 Villaviciosa de Odón, Spain; 8 Department of Neurology, Ramón y Cajal University Hospital, Red Española de Esclerosis Múltiple (REEM), Red de Enfermedades Inflamatorias (REI), 28034 Madrid, Spain; lufrossard@yahoo.es (L.C.-F.); penry02@hotmail.com (E.M.); susana.sainzdelamaza@salud.madrid.org (S.S.d.l.M.); 9 Department of Medicine, Faculty of Medicine, Alcalá de Henares University, 28805 Alcalá de Henares, Spain; 10 Department of Neurology, Gregorio Marañón General University Hospital, 28007 Madrid, Spain; marisamgines@hotmail.com; 11 Department of Neurology, Príncipe de Asturias University Hospital, 28805 Alcalá de Henares, Spain; lucia.ayuso@salud.madrid.org

**Keywords:** radiologically isolated syndrome, multiple sclerosis, quality of life, fatigue, EuroQol, FAMS

## Abstract

**Background**: Radiologically isolated syndrome (RIS) is defined by MRI findings that are suggestive of multiple sclerosis (MS) in the absence of prior clinical demyelinating events. We aimed to compare the health-related quality of life (HRQoL) between RIS and relapsing–remitting MS (RRMS) after adjusting for fatigue, cognition, and psychological distress, and to contextualize generic HRQoL, relative to healthy controls. **Methods**: In this cross-sectional analysis of the baseline data, 30 RIS, 29 RRMS, and 30 healthy controls were analyzed. MS-specific HRQoL (patients only) was assessed with the Functional Assessment of Multiple Sclerosis (FAMS), and generic HRQoL (all participants) was assessed with the EuroQol-5D (EQ-5D) visual analogue scale and utility index. Multi-variable linear regression models with robust (HC3) standard errors were used, adjusting for demographics, fatigue impact, cognitive performance, and psychological distress. **Results**: The FAMS totals were similar in RIS vs. RRMS (median 167.5 vs. 164.0; *p* = 0.694) and remained non-different after adjustment (β= −2.37, 95% CI −10.18 to 5.44; *p* = 0.544). EQ-5D outcomes showed an unadjusted gradient across groups, but adjusted differences relative to RIS were not statistically significant. Greater fatigue impact was associated with poorer HRQoL across all models (all *p* < 0.001). Psychological distress was associated with lower FAMS (β = −14.53; *p* < 0.001) but not with EQ-5D outcomes. **Conclusions**: HRQoL in RIS was comparable to RRMS, and fatigue impact was the most consistent correlate of poorer HRQoL.

## 1. Introduction

Radiologically isolated syndrome (RIS) refers to individuals with incidental magnetic resonance imaging (MRI) abnormalities characteristic of multiple sclerosis (MS), without any prior clinical demyelinating events [1]. Since it was first described, RIS has been considered an early stage on the MS spectrum and has sparked questions about prognosis, monitoring, and the timing and appropriateness of disease-modifying therapy (DMT) [2,3,4,5].

Observational cohorts show that a substantial proportion of people with RIS develop a first clinical event or meet MS diagnostic criteria over time, with risk influenced by age, sex, spinal cord or infratentorial lesions, and cerebrospinal fluid oligoclonal bands [4,6]. Proposed revisions to RIS criteria and randomized trials of early treatment (e.g., teriflunomide) have renewed discussion about RIS as a presymptomatic phase within the MS spectrum [7,8].

Beyond clinical conversion, RIS may also involve a significant patient-reported burden. Fatigue, cognitive symptoms, and psychological distress have been described in RIS and are major determinants of health-related quality of life (HRQoL) in demyelinating disorders [9,10,11,12,13,14,15,16,17]. The available studies suggest that generic HRQoL may be lower than in healthy controls and that mood and fatigue are important correlates, with variable differences between RIS and early relapsing–remitting MS (RRMS) [11,13,15,16].

However, prior work has used heterogeneous HRQoL instruments and has often relied on unadjusted comparisons or bivariate associations, leaving uncertainty about whether HRQoL differences between RIS and RRMS persist after accounting for fatigue, cognition, and psychological distress simultaneously.

To address this gap, we analyzed the baseline data from a consecutive, clinically referred cohort recruited from specialized MS units. We compared MS-specific HRQoL (Functional Assessment of Multiple Sclerosis; FAMS) [18,19,20] between RIS and RRMS and contextualized generic HRQoL (EQ-5D), relative to healthy controls [21]. We hypothesized that HRQoL in RIS would be comparable to that in RRMS after adjusting for demographics, fatigue impact (Daily Fatigue Impact Scale for Daily Use; D-FIS) [22], cognitive performance, and psychological distress.

## 2. Methods

This cross-sectional observational study is reported in accordance with the STROBE (Strengthening the Reporting of Observational Studies in Epidemiology) statement; the completed checklist is provided as Supplementary Table S1.

### 2.1. Standard Protocol Approvals, Registrations, and Patient Consents

All participants provided written informed consent, and the study was approved by the Research Ethics Committee of 12 de Octubre University Hospital, Madrid, Spain (TP16/0074; approved 20 December 2016).

### 2.2. Participants

Patients were identified from the clinical databases of specialized MS units in Madrid, Spain, and all eligible individuals were approached consecutively for participation between February 2017 and March 2021. RIS was defined as incidental MRI abnormalities that are highly suggestive of demyelination in individuals without prior clinical demyelinating events, according to the criteria in use when the cohort was initiated [1]. In 53.3% of RIS cases, the index MRI was obtained for headache. MS participants met the 2017 McDonald criteria [23]. Healthy controls were recruited from the community and hospital settings and had no history of neurological disease.

Key exclusion criteria for RIS and MS included substance abuse, major acute comorbidities, or serious chronic illness (stable chronic conditions were permitted). Healthy controls and MS participants were group-matched to the RIS group by age, sex, and years of education; the same exclusion criteria were applied to the healthy controls. All participants underwent baseline clinical evaluation and completed patient-reported outcomes and neuropsychological testing; only the baseline data were analyzed.

### 2.3. Clinical and Demographic Variables

Age, sex, and years of formal education were recorded. Disability in RRMS was assessed using the Expanded Disability Status Scale (EDSS) [24].

### 2.4. RIS Conversion-Risk Markers

For RIS participants, we recorded the presence of spinal cord lesions (cervical and/or thoracic) and cerebrospinal fluid abnormalities that are commonly associated with future clinical conversion, including an elevated IgG index (>0.7) and cerebrospinal fluid oligoclonal bands (IgG and IgM lipid-specific bands), when available from the routine diagnostic work-up. Dissemination in time on MRI was also recorded when available. Given the small sample size and the limited availability of these markers, analyses of RIS risk markers were descriptive and exploratory.

### 2.5. Health-Related Quality of Life

MS-specific HRQoL was evaluated in RIS and RRMS groups using the Spanish version of the Functional Assessment of Multiple Sclerosis (FAMS) questionnaire [18,19,20]. Items were rated on a 5-point Likert scale (0–4). The Spanish FAMS comprises 52 items covering six key domains—mobility (0–28), symptoms (0–56), emotional well-being (0–32), general contentment (0–28), thinking/fatigue (0–36), and family/social well-being (0–28)—plus an additional concerns subscale (0–28) [18,19,20]. The Spanish FAMS has shown high internal consistency across subscales and for the total score (α = 0.78–0.96) [18]. Higher scores reflect better HRQoL [18,19,20]. The total FAMS score was calculated as the sum of the six core domains, excluding the additional concerns.

Generic HRQoL was assessed in all participants with the EQ-5D descriptive system and visual analogue scale (VAS) [21]. The EQ-5D assesses mobility, self-care, usual activities, pain/discomfort, and anxiety/depression, which are combined into a utility index (1.0 indicates full health) [21]. The EQ-5D VAS ranges from 0 (worst imaginable health) to 100 (best imaginable health) [21].

### 2.6. Fatigue, Mood, and Personality/Psychological Variables

Fatigue impact was assessed using the D-FIS, an eight-item instrument designed to quantify the daily impact of fatigue in MS [22]. Total scores range from 0 to 32, with higher scores indicating greater fatigue impact.

Depressive symptoms were assessed with the Beck Depression Inventory-II (BDI-II) [25] and anxiety symptoms with the State-Trait Anxiety Inventory (STAI) [26]. Personality and broader psychopathology-related dimensions were assessed with the Personality Assessment Inventory (PAI) [27].

### 2.7. Neuropsychological Assessment

Cognition was assessed using the Brief Repeatable Battery of Neuropsychological Tests (BRB-N) [28]. Verbal learning and memory were evaluated with the Selective Reminding Test (SRT) [29,30,31]. Visuospatial learning and delayed recall were assessed with the 10/36 Spatial Recall Test (SPART) [29,31]. Attention and processing speed were assessed using the Symbol Digit Modalities Test (SDMT) and the Paced Auditory Serial Addition Test (PASAT) [28]. Phonemic verbal fluency was assessed using the Word List Generation (WLG) test [31]. Executive functioning was further assessed using the Stroop Color–Word Test [32,33] and the Controlled Oral Word Association Test (FAS) [34].

### 2.8. Statistical Analyses

Analyses were performed using Python (version 3.11) with the pandas, statsmodels, and scikit-learn libraries. Continuous variables are presented as mean ± SD when approximately symmetric and as the median [Q1–Q3] when skewed or bounded; categorical variables are summarized as *n* (%). Missing data were handled using complete-case analysis within each model.

- *Sample size considerations.*

The sample size was determined pragmatically, based on the number of eligible consecutive participants available during the recruitment period; therefore, a formal a priori sample size calculation was not performed. As a post hoc sensitivity analysis (minimum detectable effect size) for the primary between-patient comparison (FAMS total, RIS vs. RRMS), the available group sizes (approximately 30 per patient group) provided 80% power (two-sided α = 0.05; two-sample *t*-test approximation) to detect a large standardized mean difference (Cohen’s d of 0.7–0.8). Accordingly, the study was powered to detect large between-group differences, whereas smaller differences may have gone undetected.

- *Unadjusted group comparisons.*

Between-group comparisons across RIS, RRMS, and healthy controls were performed using one-way analysis of variance (ANOVA) for approximately normally distributed continuous variables, Kruskal–Wallis tests for non-normally distributed variables, and χ^2^ tests for categorical variables. Patient-only comparisons (RIS vs. RRMS) used Mann–Whitney U tests for non-normally distributed outcomes. Because several EQ-5D dimension cross-tabulations had expected cell counts < 5, EQ-5D dimensions were compared using Fisher–Freeman–Halton exact tests after dichotomization (no problems vs. any problems).

Unadjusted HRQoL analyses were conducted separately for MS-specific and generic instruments. FAMS outcomes (RIS and RRMS only) and EQ-5D outcomes (all participants) are reported as the median [Q1–Q3] and compared using the Mann–Whitney U test and the Kruskal–Wallis test, respectively.

- *Principal component analysis (PCA).*

To reduce dimensionality and mitigate multicollinearity among correlated predictors, principal component analysis (PCA) was performed separately on the cognitive and psychological variables using the correlation matrix. The number of retained components was determined based on scree plots, eigenvalues, and interpretability. Component scores were derived using regression, standardized to z-scores (mean 0, SD 1), and scaled for interpretability.

The cognitive PCA included all neuropsychological measures administered: verbal learning and memory (SRT), visuospatial learning and delayed recall (10/36 SPART), attention and processing speed (SDMT and PASAT), phonemic verbal fluency (WLG and FAS), and executive functioning/inhibitory control (Stroop Color and Word Test). Higher scores indicate better cognitive performance.

The psychological PCA included depressive symptoms (BDI-II), state anxiety (STAI), and selected PAI scales (somatic complaints, anxiety, anxiety-related disorders, depression, stress, and suicidal ideation). The first component was retained and interpreted as psychological distress, with higher scores indicating greater distress.

- *Multivariable regression models.*

Separate ordinary least squares (OLS) linear regression models were fitted for each HRQoL outcome: (i) FAMS total score (RIS+RRMS), (ii) the EQ-5D visual analogue scale (RIS+RRMS+healthy controls), and (iii) the EQ-5D utility index (RIS+RRMS+healthy controls). The study group was modeled using indicator variables, with RIS as the reference category. All models were adjusted for age, sex (female as the reference group), years of education, fatigue impact (D-FIS), cognitive performance (z-score), and psychological distress (z-score).

The model diagnostics included visual inspection of residual and Q–Q plots. Inference was based on heteroskedasticity-consistent (HC3) robust standard errors, with results reported as β coefficients, 95% confidence intervals, and two-sided *p*-values. Multicollinearity was assessed using variance inflation factors (VIF), and model fit was summarized using R^2^. Statistical significance was defined as *p* < 0.05.

## 3. Results

The sample comprised 89 participants: 30 with RIS, 29 with RRMS, and 30 healthy controls (Table 1). Groups were comparable in age, sex distribution, and education (all *p* > 0.05). Disability in RRMS was low (EDSS 2.0 [1.0, 3.0]). The fatigue impact (D-FIS) showed a borderline difference across groups (*p* = 0.051), whereas cognitive performance and psychological distress differed significantly (*p* = 0.013 and *p* = 0.014, respectively). The full set of characteristics is shown in Table 1.

Within RIS, spinal cord demyelinating lesions were documented in 9/25 participants (36%) with available spinal MRI. Cerebrospinal fluid abnormalities were common, with ≥1 abnormal CSF marker (elevated IgG index and/or oligoclonal bands) present in 14/20 (70%) with available CSF data. On-time dissemination was observed on MRI in 10/28 (36%); however, risk-stratified HRQoL analyses were not performed because subgroup sizes were small and highly imbalanced, and risk-marker data were incomplete.

**Figure 1:**
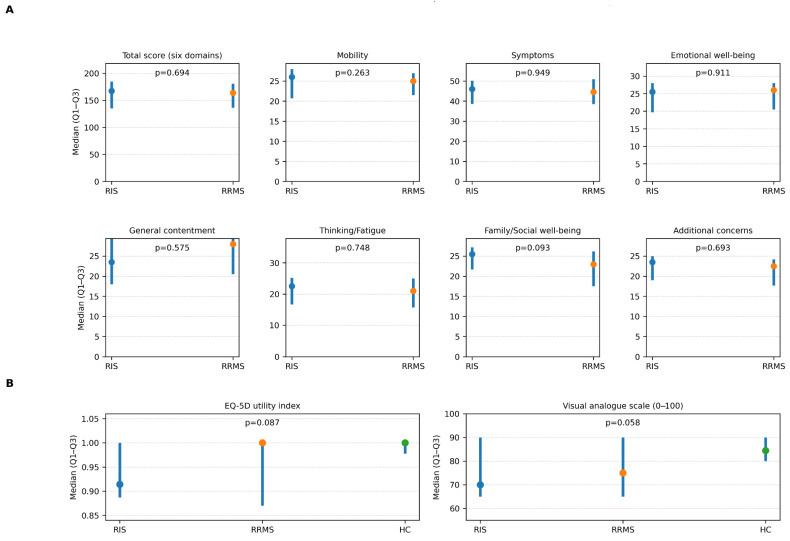
Unadjusted health-related quality of life outcomes in radiologically isolated syndrome (RIS), relapsing–remitting multiple sclerosis (RRMS), and healthy controls. (**A**) shows MS-specific HRQoL, measured with the Functional Assessment of Multiple Sclerosis (FAMS) total score (six domains) and subscales, in radiologically isolated syndrome (RIS) and relapsing–remitting multiple sclerosis (RRMS). (**B**) shows generic HRQoL measured with the EuroQol-5D (EQ-5D) utility index and EQ-5D visual analogue scale (VAS) in RIS, RRMS, and healthy controls (HC). For each outcome, points represent the median, and vertical bars represent the interquartile range (Q1–Q3). *p* values correspond to Mann–Whitney U tests for FAMS comparisons (RIS vs. RRMS) and Kruskal–Wallis tests for EQ-5D comparisons (three-group). Higher values indicate better HRQoL for all outcomes.

FAMS total scores were similar in RIS and RRMS (167.5 [135.2–185.0] vs. 164.0 [136.2–181.0]; *p* = 0.694), with no significant differences across domains or the additional concerns subscale (all *p* > 0.05), although family/social well-being showed a non-significant trend (*p* = 0.093) (Table 2; Figure 1A).

The EQ-5D VAS and utility index showed a descriptive gradient (lowest in RIS, highest in controls) without statistically significant overall differences (VAS *p* = 0.058; utility *p* = 0.087) (Table 2; Figure 1B).

Anxiety/depression and pain/discomfort were the most frequently affected dimensions, particularly in RIS. Any anxiety/depression was reported by 37.0% of RIS, 18.5% of RRMS, and 6.7% of healthy controls (*p* = 0.017), whereas pain/discomfort did not differ significantly (*p* = 0.580). Other dimensions were infrequently endorsed and did not differ between groups (mobility: *p* = 0.733; self-care: *p* = 0.535; usual activities: *p* = 0.088).

**Figure 2:**
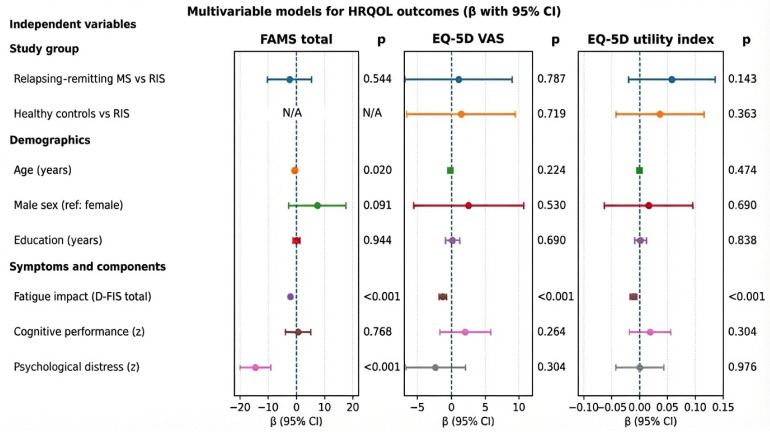
Multivariable models for health-related quality of life outcomes. Forest plot of adjusted regression coefficients (β) with 95% confidence intervals (CIs) for independent variables in the models of multiple sclerosis (MS)-specific HRQoL (FAMS total), EQ-5D VAS, and EQ-5D utility index. The vertical dashed line indicates no association (β = 0). The *p* values are shown to the right of each panel. The reference categories were radiologically isolated syndrome (RIS) for the study-group contrasts and female for sex; cognitive performance and psychological distress were standardized component scores (z). For the FAMS model, only patient groups (RIS and RRMS) were included; therefore, the healthy-control contrast was not applicable (NA).

After adjustment, RRMS did not differ from RIS (β = −2.37, 95% CI −10.18 to 5.44; *p* = 0.544). Higher fatigue impact (β = −2.04 per D-FIS point; *p* < 0.001) and greater psychological distress (β = −14.53 per SD; *p* < 0.001) were independently linked to lower FAMS, and older age was also related to lower FAMS (β = −0.60 per year; *p* = 0.020). Cognitive performance and education were not independently associated, and sex showed a non-significant trend (*p* = 0.091) (Table 3; Figure 2).

The adjusted group differences were not significant (RRMS vs. RIS *p* = 0.787; healthy controls vs. RIS *p* = 0.719). Higher fatigue impact remained independently associated with lower VAS (β = −1.21 per D-FIS point; *p* < 0.001), whereas psychological distress and cognitive performance were not (Table 3; Figure 2).

The adjusted group differences were again non-significant (RRMS vs. RIS *p* = 0.143; healthy controls vs. RIS *p* = 0.363). Fatigue impact was independently associated with a lower utility index (β = −0.011 per D-FIS point; *p* < 0.001), whereas psychological distress and cognitive performance were not (Table 3; Figure 2).

## 4. Discussion

In this cross-sectional baseline analysis, patients with RIS reported MS-specific HRQoL that was comparable to that of participants with RRMS, even after adjustment for demographics, fatigue impact, cognitive performance, and psychological distress. Generic HRQoL, assessed with the EQ-5D, showed a descriptive gradient in unadjusted analyses (lowest in RIS and highest in healthy controls), but the adjusted group differences were not statistically significant for either EQ-5D VAS or the EQ-5D utility index. Across the outcomes, fatigue impact emerged as the most consistent independent correlate of worse HRQoL. In contrast, psychological distress was independently associated with MS-specific HRQoL (FAMS) but did not remain independently associated with EQ-5D outcomes in adjusted models.

The lack of significant differences between RIS and healthy controls on the generic EQ-5D likely reflects the limited sensitivity of generic HRQoL tools to detect early, non-motor disease burden [10,17]. The EQ-5D emphasizes mobility, self-care, and usual activities—areas that are rarely affected in RIS and showed no differences between groups—limiting its ability to identify impairments mainly caused by fatigue and psychological distress [10,17]. Additionally, the EQ-5D utility index exhibits a ceiling effect, as evidenced by the high proportion of maximum scores among healthy controls and RRMS participants, which further limits its ability to distinguish at higher levels of perceived health. Conversely, MS-specific instruments, such as the FAMS, are designed to measure symptoms particularly relevant to demyelinating disease, including cognitive dysfunction, fatigue, and emotional well-being, potentially revealing a clinically meaningful burden in RIS that is not captured by generic measures [18,19,20,35]. Overall, these points suggest that the observed similarity between RIS and healthy controls on EQ-5D does not mean there is no patient-reported burden in RIS, but rather underscores the importance of disease-specific tools for assessing HRQoL early in the MS spectrum.

Our findings contribute to a growing body of evidence indicating that RIS is associated with clinically relevant symptom burden and psychosocial impact [11,13,15,16]. Taken together, these data suggest that characterizing RIS as a “benign” incidental imaging finding may be misleading when patient-reported outcomes—particularly fatigue, mood symptoms, and HRQoL—are considered.

Notably, our MS-specific findings relied on the FAMS instrument. While FAMS has been validated in MS populations [18,19,20,35], it is not routinely used in RIS. Nevertheless, the overlap between RIS and RRMS scores on an MS-specific HRQoL measure is clinically meaningful. It indicates that patient-reported domains such as thinking/fatigue, symptoms, and emotional well-being may already be compromised in RIS, as reflected by their similarity to RRMS in this cohort. The absence of statistically significant differences between RIS and RRMS does not imply identical mechanisms; rather, it may reflect the low disability burden in the RRMS group (median EDSS 2.0 [1.0–3.0]) and the predominance of non-motor determinants—particularly fatigue impact (and psychological distress for MS-specific HRQoL)—in shaping perceived health status.

Several mechanisms might explain impaired or MS-like HRQoL in RIS. First, subclinical central nervous system demyelination and network dysfunction may lead to fatigue, cognitive inefficiency, and mood symptoms, even without focal neurological deficits [36,37]. Previous studies have found cognitive deficits in RIS compared to healthy controls and, in some cases, compared to clinically isolated syndrome [12], supporting the idea of functional impact at this stage. Second, the psychological effects of receiving an uncertain diagnosis and living with the risk of MS could directly lower HRQoL, increase anxiety, and heighten symptom perception. Our models indicate that once fatigue impact and psychological distress are included, the diagnostic group itself explains little additional variance in generic HRQoL.

The current findings have practical implications for clinical care and research. Clinicians should avoid assuming that RIS is asymptomatic or benign from the patient’s perspective and should proactively assess fatigue, mood symptoms, and psychosocial stressors. Fatigue and psychological distress are potentially modifiable through patient education, sleep optimization, management of comorbidities, structured exercise, and evidence-based psychological interventions [38,39]. Moreover, as treatment trials in RIS emerge, incorporating patient-reported outcomes alongside conversion and MRI endpoints may help quantify benefits that matter to patients.

Our results also inform the broader debate regarding RIS management. While risk stratification for conversion remains central to decisions about monitoring and early disease-modifying therapy [7,8], patient-reported burden may justify supportive interventions regardless of conversion risk and may influence shared decision-making about initiating therapy. Recent discussions and trials have moved RIS from an incidental imaging descriptor toward a potential preclinical phase of MS [7,8]. Patient-centered outcomes provide an additional dimension to this evolving framework.

Several research priorities emerge. Longitudinal studies stratified by conversion status and established imaging or cerebrospinal fluid risk markers are necessary to define HRQoL trajectories in RIS. Future cohorts should also incorporate MRI measures of lesion burden and brain atrophy, along with biomarkers of neuroaxonal injury, to determine whether fatigue and psychological distress correlate with biological disease activity. Interventional studies are needed to assess whether targeted non-pharmacological approaches (e.g., fatigue management, cognitive-behavioral therapy, exercise) can improve HRQoL in RIS. As disease-modifying therapy trials progress, including patient-reported outcomes may reveal benefits or harms that are not reflected by conversion rates alone [8].

The strengths of this study include recruiting three well-characterized groups, using both disease-specific and generic HRQoL measures, and conducting detailed assessments of fatigue, neuropsychological functioning, and psychological health. To keep the model simple with a modest sample size, we reduced high-dimensional cognitive and psychological data, using PCA, and included prespecified covariates in multivariable models.

Several limitations should be acknowledged. First, the analysis is cross-sectional; therefore, causal inferences and temporal sequencing (e.g., whether fatigue precedes HRQoL impairment) cannot be established. Second, the cohort was recruited from specialized centers and may not be representative of all individuals with RIS. Because recruitment occurred in tertiary MS units, the sample reflects a consecutive clinical referral population, rather than a population-based screened cohort; referral or ascertainment bias (e.g., toward more symptomatic or health-conscious individuals) cannot be excluded. Third, although we documented selected MRI/cerebrospinal fluid conversion-risk markers, the small RIS sample size and incomplete availability of these markers precluded reliable risk-stratified or correlation analyses. Finally, although FAMS has been validated in MS populations, it has not been formally validated in RIS, and some degree of measurement non-equivalence may be present. Future studies could incorporate additional MS-specific instruments and qualitative approaches to further characterize HRQoL in RIS.

In summary, within a baseline cohort including RIS, RRMS, and healthy controls, HRQoL in RIS was like that in RRMS on an MS-specific measure and generally lower than in healthy controls on generic EQ-5D assessments. Fatigue was the most consistent independent factor linked to poorer HRQoL across outcomes, while psychological distress was independently associated with MS-specific HRQoL. These findings indicate that RIS may not be entirely benign from the patient’s perspective, and they highlight the importance of routine screening and management of fatigue and psychological distress in patients with RIS.

## Figures and Tables

**Table 1:** Baseline demographic, clinical, fatigue, cognitive, and psychological characteristics.

Variable	Radiologically Isolated Syndrome (*n* = 30)	Relapsing–Remitting Multiple Sclerosis (*n* = 29)	Healthy Controls (*n* = 30)	*p* Value
Age (years), mean ± SD	41.7 ± 8.1	41.4 ± 7.2	40.9 ± 7.8	0.925 *
Female sex, *n* (%)	26 (86.7%)	20 (69.0%)	24 (80.0%)	0.247 ^†^
Education (years), median [Q1, Q3]	15 [12–17.2]	15 [11.5–17.5]	15 [13.7–17.0]	0.662 ^‡^
Time since diagnosis (years), median [Q1, Q3]	2.5 [1.0–8.7]	4.0 [2.0–10.5]	—	0.399 **
Disability (Expanded Disability Status Scale), median [Q1, Q3]	—	2.0 [1.0–3.0]	—	—
Fatigue impact (Daily Fatigue Impact Scale total score), median [Q1, Q3]	3.0 [0.0, 13.0]	7.0 [2.0, 15.0]	2.0 [0.0, 8.2]	0.051 ^‡^
Depressive symptoms (Beck Depression Inventory total score), median [Q1, Q3]	6.5 [3.0, 13.5]	7.0 [2.7, 15.2]	6.0 [1.5, 8.0]	0.257 ^‡^
State anxiety (State-Trait Anxiety Inventory-State total score), median [Q1, Q3]	15.0 [8.0, 22.5]	14.0 [5.0, 20.0]	11.5 [5.7, 17.0]	0.240 ^‡^
Cognitive performance (component score; z), mean ± SD	−0.37 ± 1.12	−0.04 ± 0.93	0.39 ± 0.81	0.013 *
Psychological distress (component score; z), median [Q1, Q3]	−0.12 [−0.55, 0.96]	−0.10 [−0.59, 0.91]	−0.54 [−0.86, −0.14]	0.014 ^‡^

* One-way analysis of variance (ANOVA); ** Mann–Whitney U test; ^†^ chi-square test; and ^‡^ Kruskal–Wallis test.

**Table 2:** Unadjusted health-related quality of life outcomes.

Outcome	Radiologically Isolated Syndrome	Relapsing–Remitting Multiple Sclerosis	Healthy Controls	*p* Value ^†^
Functional Assessment of Multiple Sclerosis (FAMS)				
Total score (six domains), median [Q1, Q3]	167.5 [135.2, 185.0]	164.0 [136.2, 181.0]		0.694
Mobility, median [Q1, Q3]	26.0 [20.7, 28.0]	25.0 [21.5, 27.0]	—	0.263
Symptoms, median [Q1, Q3]	46.0 [38.7, 50.2]	44.5 [38.5, 51.0]	—	0.949
Emotional well-being, median [Q1, Q3]	25.5 [19.7, 28.0]	26.0 [20.5, 28.0]	—	0.911
General contentment, median [Q1, Q3]	23.5 [18.0, 30.2]	28.0 [20.5, 30.2]	—	0.575
Thinking/fatigue, median [Q1, Q3]	22.5 [16.7, 25.2]	21.0 [15.7, 25.0]	—	0.748
Family/social well-being, median [Q1, Q3]	25.5 [21.7, 27.2]	23.0 [17.5, 26.2]	—	0.093
Additional concerns, median [Q1, Q3]	23.5 [19.0, 25.0]	22.5 [17.7–24.2]	—	0.693
Generic HRQoL (EuroQol-5D)				
EQ-5D utility index, median [Q1–Q3]	0.914 [0.887, 1.000]	1.000 [0.870, 1.000]	1.000 [0.978, 1.000]	0.087
Visual analogue scale (0–100), median [Q1–Q3]	70.0 [65.0, 90.0]	75.0 [65.0, 90.0]	84.5 [80.0, 90.0]	0.058

Higher scores indicate better health-related quality of life for all outcomes. ^†^ Mann–Whitney U test (radiologically isolated syndrome vs. multiple sclerosis) and Kruskal–Wallis test (three-group comparison).

**Table 3:** Multivariable models for health-related quality of life outcomes.

Predictor	Functional Assessment of Multiple Sclerosis total β (95% CI)	*p* Value	EuroQol-5D Visual Analogue Scale β (95% CI)	*p* Value	EQ-5D Utility Index β (95% CI)	*p* Value
Relapsing–remitting multiple sclerosis vs. radiologically isolated syndrome	−2.37 (−10.18, 5.44)	0.544	1.08 (−6.86, 9.01)	0.787	0.058 (−0.020, 0.136)	0.143
Healthy controls vs. radiologically isolated syndrome	—	—	1.46 (−6.60, 9.52)	0.719	0.036 (−0.043, 0.116)	0.363
Age (years)	−0.60 (−1.09, −0.10)	0.020	−0.26 (−0.68, 0.16)	0.224	−0.001 (−0.006, 0.003)	0.474
Male sex (reference: female)	7.44 (−2.71, 17.59)	0.091	2.58 (−5.58, 10.75)	0.530	0.016 (−0.064, 0.096)	0.690
Education (years)	0.04 (−1.17, 1.25)	0.944	0.22 (−0.86, 1.29)	0.690	0.001 (−0.009, 0.012)	0.838
Fatigue impact (Daily Fatigue Impact Scale total score)	−2.04 (−2.66, −1.43)	<0.001	−1.21 (−1.76, −0.67)	<0.001	−0.011 (−0.017, −0.006)	<0.001
Cognitive performance (component score; z)	0.66 (−3.82, 5.13)	0.768	2.12 (−1.64, 5.88)	0.264	0.019 (−0.018, 0.056)	0.304
Psychological distress (component score; z)	−14.53 (−19.98, −9.08)	<0.001	−2.30 (−6.75, 2.14)	0.304	0.001 (−0.043, 0.044)	0.976

β coefficients (95% confidence intervals [CIs]) were estimated using multivariable ordinary least squares linear regression with heteroskedasticity-robust (HC3) standard errors. For categorical predictors, β indicates the adjusted mean difference relative to the reference category; for continuous predictors, β shows the adjusted change in outcome per one-unit increase in the predictor. The reference categories were RIS for the study-group contrasts and female for sex. Cognitive and psychological predictors were standardized component scores (z; mean 0, SD 1). For the FAMS model (patients only), the component scores were derived from the RIS/RRMS sample; for EQ-5D models (all participants), the component scores were derived from the full sample. Model fit: FAMS R^2^ = 0.867; EQ-5D VAS R^2^ = 0.491; EQ-5D utility index R^2^ = 0.397. Multicollinearity was low (maximum VIF ≤ 2.13).

## Data Availability

The data that support the findings of this study are available from the corresponding author upon reasonable request and subject to approval by the local ethics committee. The data are not publicly available, due to restrictions related to patient confidentiality and informed consent.

## Supplementary Materials

The following supporting information can be downloaded at: https://www.mdpi.com/article/10.3390/jcm15062184/s1, Supplementary Table S1: STROBE checklist for cross-sectional studies.
