# Supplementary material for: Health-Related Quality of Life in Radiologically Isolated Syndrome Resembles Relapsing–Remitting Multiple Sclerosis
Source: J Clin Med. 2026 Mar 13;15(6):2184. doi: 10.3390/jcm15062184 (PMC13027015; doi:10.3390/jcm15062184)
Supplement: Supplementary file 1 [file jcm-15-02184-s001.zip › jcm-4163089-supplementary.pdf]

Supplementary Table S1. STROBE checklist for cross-sectional studies

Completed checklist based on the STROBE (Strengthening the Reporting of Observational Studies in Epidemiology) Statement for cross-sectional studies.

Section/Topic	Item No.	Checklist item	Reported on page:line (or section)
**Title and abstract**	1a	Indicate the study's design with a commonly used term in the title or the abstract.	Page 1:30–31 (Abstract, Methods section line 1: "cross-sectional observational study")
**Title and abstract**	1b	Provide in the abstract an informative and balanced summary of what was done and what was found.	Page 1:29–41 (Abstract: Background, Methods, Results, Conclusion all present)
**Introduction**	2	Explain the scientific background and rationale for the investigation being reported.	Page 2:46–68 (Introduction: RIS definition, clinical significance, knowledge gaps)
**Introduction**	3	State specific objectives, including any prespecified hypotheses.	Page 2:69–75 (Introduction, last paragraph: hypothesis stated—"HRQoL in RIS would be comparable to RRMS after adjustment")
**Methods**	4	Present key elements of the study design early in the paper.	Page 2:77–79 (Methods opening: "cross-sectional observational study... STROBE statement")
**Methods**	5	Describe the setting, locations, and relevant dates, including periods of recruitment, exposure, follow-up, and data collection.	Pages 2–3:83–97 (Participants: specialized MS units in Madrid, Spain; recruitment February 2017–March 2021)
**Methods**	6a	Cross-sectional study—Give the eligibility criteria, and the sources and methods of selection of participants.	Pages 2 and 3:83–97 (Participants: clinical databases, consecutive recruitment, RIS and MS definitions, exclusion criteria detailed)
**Methods**	6b	Cross-sectional study—	Page 3:93–95 (Healthy

		For matched studies, give matching criteria and the number of participants with and without the outcome.	controls and MS participants group-matched to RIS by age, sex, and years of education)
**Methods**	7	Clearly define all outcomes, exposures, predictors, potential confounders, and effect modifiers. Give diagnostic criteria, if applicable.	Pages 3–5: RIS/MS diagnostic criteria (85–91), HRQoL definitions (99–124), fatigue/mood/cognition measures (125–141), statistical covariates (171–198)
**Methods**	8	For each variable of interest, give sources of data and details of methods of assessment (measurement). Describe comparability of assessment methods if there is more than one group.	Pages 3 and 4:110–141 (FAMS, EQ-5D, D-FIS, BDI-II, STAI, PAI, BRB-N battery—all described with scoring and validation references)
**Methods**	9	Describe any efforts to address potential sources of bias.	Pages 2 and 3:83–97 (Consecutive recruitment, frequency matching, standardized assessments)
**Methods**	10	Explain how the study size was arrived at.	Page 4:149–157 (Sample size considerations: pragmatic recruitment-based sample; post hoc power analysis provided for transparency)
**Methods**	11	Explain how quantitative variables were handled in the analyses. If applicable, describe which groupings were chosen and why.	Pages 4 and 5:158–198 (Continuous variables as mean±SD or median [Q1–Q3]; EQ-5D dichotomization rationale given; PCA z-scores described)
**Methods**	12a	Describe all statistical methods, including those used to control for confounding.	Pages 4 and 5:142–198 (Multivariable linear regression with robust HC3 standard errors; covariates specified: demographics, D-FIS, cognition PCA, psychological distress)
**Methods**	12b	Describe any methods used to examine	Page 3:102-109 (RIS conversion-risk markers

		subgroups and interactions.	recorded descriptively; no formal interaction testing due to sample size and incomplete data)
**Methods**	12c	Explain how missing data were addressed.	Page 4:146–147 (Complete-case analysis within each model stated explicitly)
**Methods**	12d	Cross-sectional study—If applicable, describe analytical methods taking account of sampling strategy.	Not applicable (no complex sampling design or weighting scheme used)
**Methods**	12e	Describe any sensitivity analyses.	Not applicable
**Results**	13a	Report numbers of individuals at each stage of study—e.g., numbers potentially eligible, examined for eligibility, confirmed eligible, included in the study, completing follow-up, and analysed.	Page 9:200–201 (Numbers analyzed at baseline: 30 RIS, 29 RRMS, 30 healthy controls. Earlier-stage screening numbers not systematically recorded)
**Results**	13b	Give reasons for non-participation at each stage.	Not reported. All eligible consecutive participants were approached; reasons for non-participation were not documented.
**Results**	13c	Consider using a flow diagram.	Not included
**Results**	14a	Provide characteristics of study participants (e.g., demographics, clinical, and social) and information on exposures and potential confounders.	Table 1; Page 5:212–213 (Demographics, EDSS, time since diagnosis)
**Results**	14b	Indicate number of participants with missing data for each variable of interest.	Not included
**Results**	15	Cross-sectional study—Report numbers of outcome events or summary measures.	Table 2 (pages 6:219–220) (HRQoL outcomes reported as medians and IQRs for all groups)
**Results**	16a	Give unadjusted estimates and, if applicable, confounder-	Table 2 (pages 6:219–220) (HRQoL outcomes reported as medians

		adjusted estimates and their precision (e.g., 95% confidence interval). Make clear which confounders were adjusted for and why they were included.	and IQRs for all groups); Table 3 (page 8: adjusted with 95% CIs); Page 7:248–258 (Covariates specified: age, sex, education, D-FIS, cognitive PCA, psychological distress)
**Results**	16b	Report category boundaries when continuous variables were categorized.	Page 4:165–166
**Results**	16c	If relevant, consider translating estimates of relative risk into absolute risk for a meaningful time period.	Not applicable (cross-sectional design; no time-to-event or relative risk estimates)
**Results**	17	Report other analyses done—e.g., analyses of subgroups and interactions, and sensitivity analyses.	Not reported
**Discussion**	18	Summarise key results with reference to study objectives.	Page 9:275–285 (Discussion opening: HRQoL comparable between RIS and RRMS; fatigue most consistent correlate)
**Discussion**	19	Discuss limitations of the study, taking into account sources of potential bias or imprecision. Discuss both direction and magnitude of any potential bias.	Page 11:357–369 (Limitations: cross-sectional design, sample size, referral bias, incomplete RIS risk markers)
**Discussion**	20	Give a cautious overall interpretation of results considering objectives, limitations, multiplicity of analyses, results from similar studies, and other relevant evidence.	Pages 10 and 11:286–369 (Results contextualized with prior literature; fatigue emphasized; clinical implications cautiously stated)
**Discussion**	21	Discuss the generalisability (external validity) of the study results.	Page 11:359–363 (Generalizability discussed: referral bias from specialized MS units may limit external validity)
**Other information**	22	Give the source of	Pages 11 and 12:377–

		funding and the role of the funders for the present study and, if applicable, for the original study on which the present article is based.	388 (Acknowledgments and Funding section: funding sources listed, role of funders stated)
